# *Huperzia serrata* Thallus as a Host-Derived Elicitor Enhances Alkaloid and Terpenoid Biosynthesis in a *Sporothrix* Endophyte: Insights from Multi-Omics

**DOI:** 10.3390/jof12100719

**Published:** 2026-09-24

**Authors:** Malong Cheng, Ying Guan, Liangfang Dai, Linlin Zhao, Yongchun Huang, Yaling Chen, Xiangdong Luo

**Affiliations:** College of Life Science, Jiangxi Normal University, Nanchang 330022, China

**Keywords:** *Huperzia serrata*, endophytic fungi, elicitor, alkaloids, terpenoids

## Abstract

Endophytic fungi from *Huperzia serrata* (*H. serrata*) are promising sources for exploring novel acetylcholinesterase (AChE) inhibitors. In this study, 111 endophytic fungal strains were isolated from wild *H. serrata*, of which 18 exhibited AChE inhibition rates exceeding 90%. Among them, an endophytic *Sporothrix* isolate (HS2-S-26) showed a 98.37% AChE inhibition rate and could produce a range of AChE inhibitors, including dauricine. Comparative metabolomics and transcriptomics between *H. serrata* thallus (HT)-treated and untreated cultures revealed that the HT elicitor reprogrammed HS2-S-26 metabolism, affecting 6708 genes and 49 secondary metabolites. Alkaloids and terpenoids were the two largest classes, containing 10 and 11 compounds, respectively. Differential metabolites and differentially expressed genes were co-enriched in 59 KEGG pathways related to the biosynthesis and metabolism of alkaloids, terpenoids, and tyrosine metabolism, etc. HT likely promoted alkaloid accumulation by upregulating the expression of tyrosine biosynthesis genes (*AROF*, *TYR1*, *GOT2*, and *ARO8*) and may also enhance the supply of dopamine, potentially contributing to dauricine accumulation. Meanwhile, HT may promote terpenoid biosynthesis via activation of core pathway genes (*MVAK2*, *GGPS1*, *FDFT1*) and inhibition of branch pathway genes (*COQ2*, *WRBA*). Through multi-omics, this study demonstrates that host thallus acts as an effective elicitor of endophytic secondary metabolism, offering insights into host–endophyte regulatory crosstalk.

## 1. Introduction

Endophytic fungi are microorganisms that inhabit different tissues of living plants without causing obvious external infection symptoms, and they share a symbiotic and coevolutionary relationship with their host [1]. Many strains have developed the ability to synthesize structurally novel and biologically diverse secondary metabolites, including alkaloids, terpenoids, flavonoids, and polyketides, many of which hold great application potential in pharmaceutical research [1]. Many plant endophytic fungi have also been reported to produce bioactive compounds identical or similar to those of their hosts. Since the isolation of the taxol-producing endophyte *Taxomyces andreanae* from *Taxus brevifolia* [2], many fungi have been found to produce valuable compounds, such as camptothecin, cinchonine, podophyllotoxin, deoxypodophyllotoxin, and vincristine [1,3,4]. In addition, endophytic fungi can also produce bioactive compounds that are absent in their host plants. For instance, *Acremonium sclerotigenum* from *Stevia rebaudiana* produces hydrazine carbothioamide derivatives with antimicrobial and anticancer properties [5], while *Alternaria* sp. from mangroves yields alterisocoumarins with anti-inflammatory effects [6]. *Cladosporium cladosporioides* from *Strelitzia nicolai* produces alternariol monomethyl ether with potent anticancer activity [7]. Increasing studies have demonstrated that the utilization of endophytic fungi for the regulation or direct biosynthesis of the target natural products has emerged as a sustainable, cost-effective, and reproducible approach [8].

*Huperzia serrata* (*H. serrata*), a traditional medicinal fern in China, has attracted extensive attention as a source of neuroactive metabolites with acetylcholinesterase (AChE) inhibitory activity, among which huperzine A (HupA) is the best-known example. However, the slow growth and reproductive difficulties of wild *H. serrata*, as well as the extremely low content of bioactive compounds including terpenoids and alkaloids, have made traditional plant-derived extraction insufficient to meet growing market demand [9,10]. Moreover, the structures of many important active compounds in *H. serrata* are very complex, making their chemical synthesis difficult, costly, and often resulting in low biological activity [10,11]. Therefore, the exploration and discovery of endophytic fungi from *H. serrata* that can produce target bioactive compounds has become one of the most promising and important strategies [8].

To date, more than 300 endophytic fungi have been isolated from *H. serrata* [9], such as the genera *Fusarium* [10], *Penicillium* [11], *Trichoderma* [12], *Colletotrichum* [13], and *Paecilomyces* [14]. These endophytes are valuable reservoirs of bioactive compounds, with over 200 alkaloids, steroids, terpenoids, and other natural products identified to date, many of which exhibit AChE inhibitory or antibacterial activities [15]. For example, the *H. serrata* endophyte *Phaeosphaeria* sp. LF5 produces polyketide derivatives with AChE inhibitory activity [16]; crude extracts from 46 *H. serrata* endophytic fungi showed significant cytotoxic and antibacterial activities; and a novel metabolite from *Penicillium panissanguineum* SZSS 4-2-2 exhibited strongly anti-inflammatory activity [17]. These findings indicate that the endophytic fungi from *H. serrata* have significant potential for development and application. However, a notable challenge in the industrial application of these fungi is their metabolic instability upon repeated subculturing. Some strains have been observed to gradually lose the ability to produce target compounds, often accompanied by the silencing of biosynthetic gene clusters after repeated subculturing [11,18]. A plausible explanation for this decline in productivity is the disruption of host–endophyte communication, as these fungi are natural inhabitants of plant tissues that may depend on host-derived signals for the activation and maintenance of their secondary metabolism [19]. Therefore, host-derived elicitors represent a promising strategy to reactivate silenced biosynthetic gene clusters to sustain or increase metabolite production.

Many studies have revealed that host-derived signals regulate endophytic fungi secondary metabolism through complementary mechanisms, from the supply of biosynthetic enzymes and transcriptional activation of silent gene clusters to chemical elicitation [18]. For instance, *Fusarium solani* requires host-supplied strictosidine synthase for full camptothecin biosynthesis [20], while *Epichloë* endophytes express their complete ergovaline gene clusters only during host colonization, remaining largely silent in axenic culture [19]. Similarly, *H. serrata* extracts act as signaling cues that enhance HupA production and stabilize activity over subcultures [21,22]. In recent years, the application of integrated multi-omic approaches has greatly accelerated the discovery of host–endophyte interaction networks. Schalamun et al. (2026) found that a silent secondary metabolite gene cluster in *Trichoderma harzianum* was induced up to 1000-fold upon host recognition and was essential for colonization [23]. Co-culture of *Sanghuangporus vaninii* with its native endophyte *Fusarium solani* MF20 dramatically enhanced flavonoid, terpenoid, and polysaccharide production, with integrated omics linking this overproduction to Ca^2+^ signaling, ATP accumulation, and transcriptional upregulation of terpenoid/flavonoid pathways [24]. These multi-omic studies demonstrate that host-derived signals reprogram fungal transcription and metabolism, providing a mechanistic framework for understanding host–endophyte interactions and guiding elicitor-based pathway activation.

Although hundreds of endophytic fungi have been isolated from *H. serrata* to date, their industrial application remains limited. Some strains would gradually lose the ability to synthesize target metabolites once separated from their hosts, yet the molecular mechanisms underlying this metabolic instability, particularly how host-derived signals regulate fungal secondary metabolism at the transcriptional and metabolic levels, remain largely unexplored. In this study, endophytic fungi from wild *H. serrata* were further isolated and screened for strong AChE inhibitory activity, followed by co-fermentation with *H. serrata* thallus (HT) as an elicitor. We hypothesized that HT-derived signals would activate specific biosynthetic gene networks in the endophytic fungus, leading to enhanced production of bioactive metabolites. To test this hypothesis, integrated transcriptomic and metabolomic analyses were conducted to elucidate the molecular regulatory networks and mechanisms underlying HT-induced metabolic activation. These results would lay an important foundation for the research and application of the interaction between endophytes of *H. serrata* and their host.

## 2. Materials and Methods

### 2.1. Isolation of Endophytic Fungi from Wild H. serrata

The wild *H. serrata* plants were collected from Nanping, Fujian Province, China, and identified by Professor Tu Yisheng from Jiangxi Normal University. Endophytic fungi were isolated from the healthy roots, stems, and leaves of *H. serrata* according to the previous method [9,11] with slight modification. Briefly, fresh plant samples were rinsed sequentially with tap water and sterile distilled water, then divided into roots, stems, and leaves. Surface sterilization was performed using 75% ethanol (45 s), 2% sodium hypochlorite (3 min), and 10% hydrogen peroxide (8 min), followed by washing four times with sterile water and drying with sterile filter paper. The tissue fragments were placed onto the potato dextrose agar (PDA) plates supplemented with 100 μg/mL penicillin to inhibit bacterial growth, with final rinse water as a control. Plates were incubated at 28 °C in darkness. No microbial growth was observed on these control plates, confirming the effectiveness of the surface sterilization procedure. Endophytic fungi were purified by hyphal tip transfer, stored at 4 °C (PDA slants) and −80 °C (15% glycerol). All isolated endophytic fungi were preserved at Jiangxi Normal University (Nanchang, China), and the strain used in this study (HS2-S-26) has been assigned the institutional accession number JXNU-HS2S26.

### 2.2. Identification of Endophytic Fungi

The endophytic fungi were inoculated onto PDA medium at 28 °C for 6–8 days. The preliminary identification of endophytic fungi was based on the morphology of colonies, including size, shape, color, as well as mycelia and conidia observed by light microscope. Moreover, genomic DNA of fungi was extracted using a modified cetyltrimethylammonium bromide (CTAB) method [25]; after that, the internal transcribed spacer (ITS) region of the ribosomal DNA (ITS1-5.8S-ITS2) was amplified using the universal primers ITS1 (5′-TCCGTAGGTGAACCTGCGG-3′) and ITS4 (5′-TCCTCCGCTTATTGATATGC-3′) [11]. The PCR amplification was performed using a 50 μL reaction system: template (genome) 1.4 μL, 2 × Magic Green Taq Super Mix (Tolo Biotech Co., Ltd., Shanghai, China) 25 μL, primers (10 μmol/L) each 1 μL, and water to 50 μL. The PCR protocol was as follows: initial denaturation at 95 °C for 3 min, followed by 33 cycles of 95 °C for 30 s, 55 °C for 30 s, 72 °C for 45 s, and a final extension at 72 °C for 7 min. PCR products were detected using 1% agarose gels and sequenced by Sangon Biological Engineering Co., Ltd. (Shanghai, China).

The obtained fungal ITS sequences were identified via using NCBI BLAST web service (https://blast.ncbi.nlm.nih.gov/Blast.cgi; accessed on 15 May 2024). For enhanced phylogenetic resolution, the *rpb2* (RNA polymerase II second largest subunit) gene sequence was extracted from the HS2-S-26 transcriptome assembly and subjected to BLASTn against NCBI GenBank, with the top 15 hits retrieved as references for phylogenetic analysis [26]. Phylogenetic and molecular evolutionary analyses were conducted using MEGA version 11 [27]. For phylogenetic analysis, both Neighbor-Joining (NJ) and maximum likelihood (ML) methods were employed, with 1000 bootstrap replicates [28]. The resulting trees were visualized using the online tool ChiPlot (https://chiplot.online, accessed on 15 May 2024).

### 2.3. Fungal Fermentation and Crude Extract Preparation

To prepare the seed culture for large-scale cultivation, the purified and identified endophytic fungal strains were cultured in 100 mL of potato dextrose broth (PDB) liquid medium at 28 °C with shaking at 160 rpm for 2–4 days. After that, the seed culture was individually inoculated into a 500 mL shake flask containing 200 mL of PDB at a 1% (v/v) ratio, and three replicates per treatment were set up. Subsequently, fermentation was carried out at 28 °C and 160 rpm for 14 days. The mycelia and the culture broth were separated by vacuum filtration. The fermentation broth was collected and extracted with ethyl acetate to obtain the crude extract. The mycelia were rinsed 3 times with 0.01 M phosphate-buffered saline (PBS, pH 7.4), dried at 40 °C to a constant weight, and ground to powder. For methanol extraction, dried fungal powder was subjected to three extractions using 150 mL of methanol as described by Wu et al. [29], followed by ultrasound-assisted extraction for 90 min at temperatures not exceeding 40 °C. After concentration under vacuum, the methanol extract and ethyl acetate extract were combined and then air-dried. The crude extracts were reconstituted in chromatographic-grade methanol to a final concentration of 5 mg/mL, followed by filtered through 0.22 μm membrane filters. The resulting extract was stored at −20 °C for subsequent experiments.

Additionally, HT regenerated and maintained in our laboratory [29] was harvested, thoroughly washed with sterile distilled water, and homogenized using a sterile blender. The resulting homogenate was used directly as an elicitor and added to the culture medium under aseptic conditions at final concentrations of 0 g, 1 g, 2 g, and 3 g/100 mL (based on fresh weight). The four groups were designated as S0, S1, S2, and S3, each with three replicates. HT was added at the time of fungal inoculation (day 0), and the co-cultures were incubated at 28 °C in darkness for 14 days prior to metabolite extraction.

### 2.4. Inhibition Assay of AChE Activity

The AChE inhibitory activity of the fungal extracts was determined according to Ellman’s method with some modifications [9,11]. HupA (0.02 mg/mL) was used as a positive control to validate the assay system. All fungal crude extracts were tested at a screening concentration of 1 mg/mL. The reaction mixture, with a total volume of 165 μL, consisted of 35 μL of 0.05 M phosphate buffer (pH 7.8), 30 μL of 0.2 U/mL AChE, 80 μL of 1 mM dithiobisnitrobenzoic acid (DTNB), and 20 μL of the tested sample. The mixture was prepared in a 96-well plate and incubated at 37 °C for 20 min. Then 35 μL of 7.5 mM acetylthiocholine iodide (ATCI) was added to the mixture and incubated again for 20 min at 37 °C. Subsequently, the absorbance of the mixture at 412 nm was measured using a microplate reader. For the controls, 30 μL of 0.2 U/mL AChE or 20 μL of tested sample was replaced with a similar volume of 0.05 M phosphate buffer (pH 7.8). At the same time, the HupA standard was used as a positive control. All assays were performed in triplicate, and the percent inhibition was calculated as follows: I (%) = 100% × (Absorbance of control − Absorbance of sample)/(Absorbance of control−Absorbance of blank).

### 2.5. HPLC Analysis

The HPLC analysis of crude extracts was performed using a Waters 1525 HPLC system (Waters Corporation, Milford, MA, USA) equipped with a Waters 2998 UV-Vis diode array detector monitored from 210 to 400 nm. Separation was achieved using a Waters Xterra MS C18 column (250 × 4.6 mm, 5 μm) maintained at 25 °C. The mobile phase consisted of 0.08 M ammonium acetate (pH 6.0) and methanol in a ratio of 70: 30 (*v*/*v*), and the flow rate was set at 0.8 mL/min. The effect of HT on metabolite accumulation in endophytic fungi was also preliminarily assessed via HPLC analysis of crude extracts. HupA standard (Aladdin Biochemical Technology Co., Ltd., Shanghai, China) and dauricine (Baicaoyuan Biotechnology Co., Ltd., Jiangxi, China) were used as controls to confirm their presence. A standard curve was constructed using dauricine reference standard at concentrations ranging from 0.0125 to 0.4 mg/mL for quantification. A linear relationship (R^2^ = 0.999) was observed between dauricine concentration and the peak area.

### 2.6. Metabolome Analysis by UPLC-MS/MS

For metabolomic profiling, samples were prepared exclusively from fungal mycelia, with three replicates per treatment group (control and S1). Mycelia of HS2-S-26 cultured with or without HT were collected, thoroughly washed three times with sterile PBS (pH 7.4) to minimize the carryover of extracellular HT residues, and then vacuum freeze-dried and ground into powder. About 50 mg of lyophilized powder was extracted with 1200 μL of −20 °C pre-cooled 70% methanol by vortexing for 30 s every 30 min, for a total of 6 times. After centrifugation at 12,000 rpm for 3 min, the supernatant was filtered through a 0.22 μm microporous membrane. The extract samples were examined by a Waters (Milford, MA, USA) ACQUITY UPLC system equipped with an ACQUITY Premier HSS T3 Column (1.8 µm, 2.1 × 100 mm). The injection volume was 4 μL, and gradient elution was performed for 10 min using 0.1% formic acid in water (solvent A) and 0.1% formic acid in acetonitrile (solvent B). The gradient procedure was as follows: 5–20% B (0–2 min), 60% B (2–5 min), 99% B (5–6 min), hold at 99% B (6–7.5 min), return to 5% B (7.5–7.6 min), and hold at 5% B (7.6–10 min). The flow rate was 0.4 mL/min, and the column temperature was 40 °C. MS/MS data were acquired in information-dependent acquisition (IDA) mode using Analyst TF 1.7.1 Software (Sciex, Concord, ON, Canada). The parameters were set as follows: ion source gas I, gas II, and curtain gas set at 50, 50, and 25 psi; source temperature at 550 °C; an ion spray voltage floating (ISVF) of 5000 V (positive ion mode)/−4000 V (negative ion mode). Declustering potential and collision energy were optimized, and mass scanning ranged from 25 to 1000 *m*/*z*. Metabolite annotation was performed following the Metabolomics Standards Initiative (MSI) reporting guidelines, with confidence levels assigned based on the matching criteria used.

Unsupervised principal component analysis (PCA) and orthogonal partial least-squares discriminant analysis (OPLS-DA) were performed using the R package MetaboAnalystR [30]. The model quality was assessed by the cumulative values of R^2^X, R^2^Y, and Q^2^. A permutation test with 200 random permutations was conducted to validate the model and exclude overfitting. Differential accumulated metabolites (DAMs) were screened with thresholds of variable importance in projection (VIP) values (VIP > 1) from OPLS-DA and absolute log_2_ fold change (FC) values (|log_2_FC|) ≥ 1. The DAMs were annotated and mapped to Kyoto Encyclopedia of Genes and Genomes (KEGG) pathways.

### 2.7. RNA Extraction and Transcriptome Analysis

Total RNA was extracted using the PBIOZOL Extraction Kit (BioFlux, Hangzhou, China) according to the manufacturer’s instructions. For transcriptomic analysis, three replicates were prepared for each treatment group (control and S1). RNA concentration and quality were assessed using a Qubit 4 Fluorometer (Thermo Fisher Scientific, Waltham, MA, USA) and a Qsep400 biofragment analyzer (BiOptic, Inc., Taiwan, China). mRNA was enriched from total RNA using Oligo(dT) magnetic beads, fragmented, and reverse-transcribed into double-stranded cDNA. The double-stranded cDNA underwent end-repair, A-tailing, adapter ligation, and PCR amplification. The PCR products were then circularized to generate single-stranded circular DNA libraries, followed by phi29 amplification to produce DNA nanoballs (DNBs), which were sequenced on the DNBSEQ platform (MGI) with 150 bp paired-end reads. Additionally, portions of the synthesized cDNA were stored at −80 °C for subsequent quantitative real-time PCR (qRT-PCR) assays.

Raw reads were cleaned by removing adapters, ambiguous bases (N), and low-quality reads (Q ≤ 20) using FastP [31]. Clean reads were assembled de novo with Trinity [32], then clustered and de-redundanted using Corset to obtain unigenes (the longest sequence per cluster). Unigenes were aligned with KEGG, NCBI Protein Nonredundant (NR), Swiss-Prot, the translation of EMBL (TrEMBL), Clusters of orthologous groups for eukaryotic complete genomes (KOG), and Gene Ontology (GO) databases using DIAMOND software (v2.0.9), and amino acid sequences were blasted in the Pfam database using the HMMER software (v3.3.2) to obtain the annotation information. The fragments per kilobase of exon model per million mapped fragments (FPKMs) were calculated using RSEM software (v1.3.1) to estimate gene expression levels and transcript abundances [33]. For differential expression analysis, raw count data were used as input for DESeq2 software (v1.22.2) [34], with a threshold of |log_2_FC| ≥ 1 and False Discovery Rate (FDR) < 0.05. KEGG pathway analysis was performed by TBtools 2.333 [35], with pathways of *p* < 0.05 considered significantly enriched.

### 2.8. Combined Transcriptome and Metabolome Analyses

The DAMs and differentially expressed genes (DEGs) in the S1 vs. S0 group were mapped to the KEGG pathway database to identify the shared pathways. The Pearson correlation coefficients (PCC) were calculated using the cor function in R. Correlation analyses were performed using six samples (three biological replicates per treatment group). *p*-values were adjusted for multiple testing using the Benjamini–Hochberg false discovery rate (FDR) method, and correlations with an adjusted *p* < 0.05 were considered statistically significant. For global correlation analysis, PCC > 0.8 and *p* < 0.05 were used as the significance threshold, whereas a stricter threshold (PCC > 0.8, *p* < 0.01) was applied for pathway network visualization to minimize visual complexity. The network connecting DEGs and DAMs was drawn using ChiPlot (https://www.chiplot.online/, accessed on 15 May 2024).

### 2.9. qRT-PCR Analysis for DEG Validation

The expression levels of nine DEGs were verified by qRT-PCR. Amplification was carried out using the BlasTaq™ 2×qPCR MasterMix Kit (ABM, Vancouver, BC, Canada), with β-tubulin selected as the internal reference gene. Target gene primers were designed using Primer Premier 5 (Premier Biosoft, San Francisco, CA, USA) and are listed in Table 1. The 20 μL reaction mixture contained 2 μL of cDNA (≤100 ng/reaction), 1 μL of each primer, 10 μL of the BlasTaq™ 2 × qPCR MasterMix (Before use, 11 μL of QN ROX Reference Dye was supplemented per 1.25 mL of BlasTaq™ 2 × qPCR MasterMix), and 6 μL of RNase-free water. The qRT-PCR cycling protocol was as follows: denaturation at 95 °C for 3 min, followed by 40 cycles of 95 °C for 15 s and then 60 °C for 1 min. All experimental reactions were performed in triplicate, and the relative expression level of each gene was calculated using the 2^−∆∆Ct^ method [36].

## 3. Results

### 3.1. Identification and Phylogenetic Analysis of Endophytic Fungi

A total of 111 cultivable endophytic fungi were isolated via tissue separation. The highest abundance was observed in stems (89 strains, 80.18%) and leaves (20 strains, 18.01%), revealing an obvious tissue-specific distribution pattern of endophytes in *H. serrata*. Based on morphological observation and ITS sequence analysis, 75 out of the 111 endophytic fungal strains were identified at the genus level, belonging to 3 phyla and 29 genera (Figure 1A, Table S1). The phylogenetic tree exhibited strong bootstrap support for the major clades; most major clades received moderate to strong bootstrap support (≥70%), and several genus-level clusters received bootstrap support exceeding 90%. The three phyla identified were Ascomycota, Basidiomycota, and Mucoromycota, with Ascomycota being the dominant phylum (93 strains, 83.78%). At the genus level, *Colletotrichum* (23 strains, 20.72%) and *Podospora* (10 strains, 9.01%) were the two most predominant endophytes. The remaining 36 strains could not be assigned to a specific genus, and most of them were classified as Sordariomycetes (22strains, 19.82%) and Dothideomycetes (10 strains, 9.01%). Additionally, to improve taxonomic resolution, we extracted the *rpb2* gene sequence from the transcriptome assembly of HS2-S-26 and constructed a phylogenetic tree based on *rpb2* (Figure S1). The tree placed HS2-S-26 within the genus *Sporothrix* with strong bootstrap support (100%), clustering with reference strains of *S. schenckii* and *S. globosa*. However, the sequence identity between HS2-S-26 and the closest reference strain was approximately 80% (Table S2), which is below the threshold typically expected for species-level assignment. This suggests that HS2-S-26 may represent a novel or previously uncharacterized species within the genus *Sporothrix*. The fungal colonies exhibit different colors and shapes as shown in Figure 1B. Light microscopy revealed morphological differences among the isolates, with most showing branched hyphae and a minority displaying sporangia and spores (Figure S2). These morphological characteristics suggest a broad taxonomic diversity among the isolated endophytic fungi.

### 3.2. AChE Inhibition and HupA Detection of Fungal Extracts

To avoid redundant screening, 1–2 representative strains were randomly selected from each identified species of fungi for the AChE inhibitory activity assessment, ultimately resulting in a final total of 65 strains. The results demonstrated that all fungal strains exhibited AChE inhibitory activity to varying degrees (Table S3). Among the 65 strains tested, 18 strains (27.69%) showed AChE inhibition rates exceeding 90%, and 6 strains exhibited inhibition rates higher than that of the positive control HupA (95.14%) under the assay conditions tested. An additional 13 strains (20.00%) exhibited inhibition rates of 80–90%, and 20 strains (30.77%) demonstrated moderate inhibition (50–80%). The remaining 14 strains (21.54%) showed inhibition rates below 50%. The crude extracts of the 51 fungal strains with an AChE inhibition rate exceeding 50% were examined for the presence of HupA by HPLC analysis. The results showed that HS2-S-26 exhibited a chromatographic peak showing a retention time similar to the HupA standard at 308 nm (Figure S3). However, due to its relatively low content, further identification is needed to determine whether this endophytic fungus can produce HupA.

### 3.3. Effects of HT on Metabolite Accumulation in HS2-S-26

Recent studies have emphasized the critical role of elicitors, particularly the host plant itself, in enhancing metabolite diversity and production in endophytic fungi [8,37]. To explore HT effects on metabolite accumulation, HS2-S-26 was selected for its high AChE inhibitory activity (98.37%) and putative HupA-producing capacity (Table S3), treated with different HT concentrations, and the fermentation products were subsequently extracted for analysis. Multi-wavelength HPLC analysis revealed that 1–3 g/100 mL HT significantly increased UV-Vis-absorbing substance content in the crude extracts, with all treated groups showing higher levels than the untreated control (Figure 2). Compared to other groups, the S1 group showed stronger UV-Vis absorption and a higher density of red feature peaks indicative of high-abundance metabolites, suggesting that S1 more effectively promotes substantial accumulation of metabolites. These results indicate that an optimal concentration of HT elicitor (1 g/100 mL) would significantly promote metabolite biosynthesis in HS2-S-26.

### 3.4. Metabolomic Differences Between S1 and S0

To elucidate the molecular mechanism by which host-derived elicitors enhance secondary metabolite production in the fungus, S1 and S0 (showing the most pronounced differences) were selected for non-targeted metabolomic. PCA revealed that PC1 and PC2 explained 52.01% and 14.64% of the total variance, respectively, indicating good intra-group repeatability and inter-group differences (Figure S4). The OPLS-DA model yielded R^2^X(cum) = 0.638, R^2^Y(cum) = 1, and Q^2^(cum) = 0.951, indicating strong explanatory and predictive capacity. A permutation test with 200 random permutations confirmed the robustness of the model, and the model was not overfitted (Figure S5). UPLC-MS/MS detected 944 metabolites in the S0 and S1 groups, among which 286 were identified as DAMs (|log_2_FC| ≥ 1, VIP ≥ 1), including 158 upregulated and 128 downregulated. These DAMs were assigned to 14 distinct categories, encompassing amino acids and derivatives (64), lipids (58), organic acids (33), benzene and substituted derivatives (20), nucleotides and derivatives (17), alkaloids (13), terpenoids (12), phenolic acids (9), heterocyclic compounds (6), flavonoids (6), lignans and coumarins (5), steroids (2), quinones (2), and others (39) (Table S4). These DAMs exhibited distinctly different abundance patterns between the two groups (Figure 3A).

Of the 286 DAMs, 49 were identified as known secondary metabolites from plant endophytic fungi, including steroids, alkaloids, phenolic acids, flavonoids, lignans, coumarins, quinones, and terpenoids [1,38]. Among these, 39 were upregulated and 10 downregulated, with alkaloids and terpenoids representing the predominant metabolites (Figure 3B). The majority (10 out of 13) of the significantly differential alkaloids were upregulated (Table 2). Except for veranisatin B, all differentially accumulated terpenoids were upregulated. Furthermore, KEGG enrichment analysis mapped the differential metabolites to 86 pathways. Of these, the differential alkaloid and terpenoid secondary metabolites were specifically enriched in pathways including ubiquinone and other terpenoid-quinone biosynthesis (ko00130), terpenoid backbone biosynthesis (ko00900), diterpenoid biosynthesis (ko00904), arginine and proline metabolism (ko00330), tyrosine metabolism (ko00350), and tryptophan metabolism (ko00380) (Figure 3C).

### 3.5. Transcriptomic Differences Between S1 and S0

Meanwhile, the same S0 and S1 samples were subjected to transcriptomic sequencing to complement the metabolomic profiles. After filtering out low-quality reads, clean reads of at least 6 Gb were obtained for all samples, with Q20 and Q30 values both exceeding 92% (Table S5). These indices demonstrate the high quality of the sequencing data. A total of 19,329 unigenes were obtained from the assembly of high-quality clean reads, with most being longer than 1000 bp (Figure S6). A total of 18,556 genes (96.00%) were annotated in at least one of these databases (Figure S7, Table S6).

Differential genes were identified utilizing the criteria of FDR < 0.05 and |log_2_FC| ≥ 1. A total of 6708 DEGs were detected in S1 vs. S0, comprising 3449 upregulated and 3259 downregulated genes in S1. These DEGs were annotated into 137 KEGG pathways (Table S7). Notably, 98 of these pathways were related to metabolism, among which enriched pathways included tyrosine metabolism (ko00350), tryptophan metabolism (ko00380), phenylalanine, tyrosine and tryptophan biosynthesis (ko00400), terpenoid backbone biosynthesis (ko00900), diterpenoid biosynthesis (ko00904), sesquiterpenoid and triterpenoid biosynthesis (ko00909), ubiquinone and other terpenoid-quinone biosynthesis (ko00130), etc. Notably, the pathways enriched in the transcriptome were highly consistent with those of the metabolome, commonly involving alkaloid (ko00350, ko00380) and terpenoid (ko00130, ko00900, and ko00904) biosynthesis pathways.

### 3.6. Correlation Analysis of Differential Alkaloid and Terpenoid Metabolites with DEGs

In this study, alkaloids and terpenoids were the most significantly altered metabolite classes, with some compounds exhibiting AChE inhibitory activity [39,40,41]. Integrated transcriptomic and metabolomic analysis revealed that 6708 DEGs and 286 DAMs were co-enriched in 59 KEGG pathways (Figure S8, Table S8), including five pathways involved in alkaloid and terpenoid biosynthesis (Figure 4A). Considering that metabolites from the phenylalanine, tyrosine, and tryptophan biosynthesis (ko00400) pathway serve as vital precursors for alkaloid and terpenoid biosynthesis, DEGs associated with this pathway were pooled together for comprehensive analysis. A total of 163 unigenes were enriched in the six pathways, encoding 48 enzymes (Table S9). Pearson correlation analysis was further performed between 25 differential metabolites (alkaloids and terpenoids) and their biosynthetic DEGs (Figure 4B). The results revealed that 45 unigenes encoding 19 enzymes were significantly positively correlated (PCC > 0.8, *p*< 0.01) with 19 DAMs (Figure 4C, Tables S10 and S11). Among them, 29 unigenes (encoding 17 enzymes) were associated with 8 alkaloid metabolites, 39 unigenes (encoding 16 enzymes) with 11 terpenoid metabolites, and 23 unigenes (encoding 14 enzymes) with both metabolite classes.

Correlation analysis revealed significant positive associations between multiple key enzyme-encoding genes and the accumulation of both alkaloid and terpenoid metabolites. Genes from the *4CL* (4-coumarate-CoA ligase), *COQ2* (4-hydroxybenzoate polyprenyl-transferase), *HGD* (homogentisate 1, 2-dioxygenase), *GGPS1* (geranylgeranyl diphosphate synthase type III), and *TRP* (tryptophan synthase) families exhibited broad-spectrum positive correlations with both metabolite classes (1–8 compounds per family), suggesting they may serve as pleiotropic regulators across both pathways (Table S11). Additionally, the *FAHD1* (acylpyruvate hydrolase) family exhibited functional divergence: two clusters were associated with alkaloids, while four were linked to terpenoids. More restricted correlation patterns were observed for *ALDH* (aldehyde dehydrogenase, NAD^+^), *ALDH8A1* (aminomuconate-semialdehyde dehydrogenase), and *INDO* (indoleamine 2,3-dioxygenase) clusters, each correlating with a single alkaloid. These results indicate that alkaloid and terpenoid biosynthesis in HS2-S-26 is a complex process regulated by multiple coordinated genes.

### 3.7. Gene Expression Patterns Related to Dauricine and Terpenoid Biosynthesis

UPLC-MS/MS analysis revealed that among the 10 upregulated alkaloids with the most pronounced changes in content, dauricine exhibited the strongest AChE inhibitory activity and the highest pharmaceutical value. Its characteristic ion at [M-H]^−^ = *m*/*z* 623.3024 is shown in Figure 5A,B, with higher accumulation observed in S1 than in S0 (Table S4). HPLC quantification also showed that dauricine content was higher in S1 than in S0, with a yield of 0.219 mg/L in S0 (Figure 5C,D). Thus, we focused on the regulatory mechanism of dauricine biosynthesis. Dauricine is a typical isoquinoline alkaloid, which is biosynthetically derived from tyrosine via the intermediates tyramine and dopamine [42,43,44]. Integrative analysis showed significant differential expression of tyrosine biosynthesis pathway genes in the S1 group, with a significant increase in dopamine (log_2_FC = 1.10). HT treatment significantly upregulated the expression of synthase genes in the tyrosine biosynthesis pathway, including *AROF* (3-deoxy-7-phosphoheptulonate synthase), *AROB* (3-dehydroquinate synthase), *TYR1* (prephenate dehydrogenase, NADP+), *GOT2* (aspartate aminotransferase, mitochondrial), and *ARO8* (aromatic amino acid aminotransferase I/2-aminoadipate transaminase) (Figure 6). These results suggest that HT may promote dauricine accumulation through the tyrosine to dopamine pathway.

To better understand the regulatory mechanism underlying HT-induced terpenoids biosynthesis and accumulation, we further analyzed the expression profiles of genes involved in the terpenoid biosynthetic network. Transcriptomic annotation revealed that terpenoid backbone biosynthesis in HS2-S-26 solely relies on the intact mevalonate (MVA) pathway (Figure 6). This pathway involves multiple key enzyme genes, including *HMGCS* (3-hydroxy-3-methylglutaryl-CoA synthase), *HMGCR* (3-hydroxy-3-methy-lglutary-CoA reductase), *MVAK1* (mevalonate kinase), *MVAK2* (phosphomevalonate kinase), *GGPS1*, *FDFT1* (farnesyl-diphosphate farnesyltransferase), and *SQLE* (squalene monooxygenase), which sequentially convert acetyl-CoA into isopentenyl diphosphate (I-PP), the universal precursor of terpenoids. *GGPS1* catalyzes the conversion of I-PP (isopentenyl diphosphate) into G-PP (geranyl diphosphate), F-PP (farnesyl-diphosphate), and GG-PP (geranyl geranyl diphosphate), which serve as precursors for monoterpenoids, sesqui-/triterpenoids, and diterpenoids, respectively. Heatmap comparison showed that most MVA pathway gene clusters were significantly upregulated in S1 compared to S0, with gene family members exhibiting divergent expression patterns that coordinately drove the simultaneous biosynthesis of diverse terpenoids. Among these, the top two upregulated putative terpenoid synthase genes were annotated as *MVAK2* (Cluster-8440.12; log_2_FC = 5.29) and *FDFT1* (Cluster-8157.7; log_2_FC = 3.03), encoding phosphomevalonate kinase and farnesyl-diphosphate farnesyl-transferase, respectively. *MVAK2* participates in the MVA pathway to supply core precursors for terpenoid backbone biosynthesis, while *FDFT1* acts downstream of the terpenoid backbone pathway and specifically functions in triterpenoid biosynthesis.

qRT-PCR validation of nine selected genes (*HMGCR*, *AROB*, *GGPS1*, *GOT2*, *FDFT1*, *MVAK1*, *HMGCS*, and two other gene clusters) showed expression profiles largely consistent with the transcriptome data (Figure S9), reinforcing the reliability of the RNA-Seq analysis. Thus, we propose that the increase in dauricine levels is associated with the activation of the tyrosine pathway and subsequent dopamine accumulation, while the elevation of terpenoid levels is likely linked to the upregulation of the terpenoid backbone pathway and enhanced precursor availability.

## 4. Discussion

Endophytic fungi in medicinal plants exhibit rich diversity and versatile bioactive natural products, serving as precious reservoirs for biotechnological and medical exploitation, and showing vital significance and broad application potential in natural drug development. Previous data indicated that among the 15 to 30 new pharmaceuticals launched in the United States each year, nearly 75% of drug candidates are mainly derived from microbial origins rather than plant synthetic pathways [45]. In this study, endophytic fungal strains of *H. serrata* were isolated and classified into 3 phyla and at least 29 genera, reflecting a significant endophytic fungal diversity that can serve as an abundant resource for mining novel natural products. Consistent with earlier studies [46,47], our results showed that the class Sordariomycetes and the genus *Colletotrichum* were the most dominant groups among the endophytic fungi isolated from *H. serrata*.

In this study, the AChE inhibitory activity of 18 endophytic fungal strains exceeded 90%, among which 6 strains showed inhibition rates higher than that of the positive control (HupA). Metabolomic analysis revealed that the strain HS2-S-26 can synthesize several bioactive metabolites belonging to alkaloids, terpenoids, and flavonoids. Among these, dauricine, paeoniflorin, α-mangostin, and forskolin have been reported to exhibit AChE inhibitory activity [48,49,50,51]. Although HupA was not detected by UPLC-MS/MS, this strain exhibited potent AChE inhibitory activity, suggesting that its pharmaceutical potential may be derived from alternative metabolites rather than HupA itself. It should be noted, however, that the present study is a preliminary screening, and the IC_50_ values of the active strains were not determined. Future quantitative evaluation of IC_50_ would enable a more precise comparison of their inhibitory efficacy and facilitate strain prioritization. Nevertheless, the preliminary findings suggested that HS2-S-26 represents both a valuable fungal resource for AChE inhibitor discovery and a unique system for studying endophyte–host regulation of natural product biosynthesis.

Under standard culture conditions, endophytic fungi lose exposure to their specialized host microenvironment, which may lead to the silencing of partial secondary metabolite biosynthetic genes [52]. Many previous studies have applied crude or water extracts from host plants as elicitors to enhance the production of specific metabolites in endophytic fungi [21,22]. For instance, the addition of *H. serrata* extracts significantly upregulated HupA biosynthesis in *Colletotrichum kahawae* [37], and co-culture of an endophytic fungus from *Dioscorea nipponica* with the ethanol extract of its host rhizome significantly promoted saponin accumulation [53]. However, the extraction and sterilization during elicitor preparation often degrade important ingredients and reduce induction efficiency. Therefore, the grinding solution of sterile fresh HT was used as the elicitor in the present study, which effectively retained major compounds and potentially improved the induction efficacy.

Elicitor-induced metabolic reprogramming has been observed in related fungal species via multi-omic approaches. He et al. (2025) demonstrated that traditional Chinese herbal powders enhanced terpenoid and triterpenoid accumulation in *Taiwanofungus gaoligongensis* and identified potential rate-limiting enzyme genes in the mevalonate pathway [54]. Similarly, integrated multi-omics of the *Ginkgo biloba* endophyte *Fusarium* sp. revealed that host-derived ginkgolic acid stress triggered comprehensive metabolic reprogramming, illustrating the utility of this approach for elucidating fungal adaptive responses to host signals [55]. Multi-omic studies of *Trichoderma harzianum* further showed that host recognition strongly upregulates a previously uncharacterized secondary metabolite gene cluster critical for rhizosphere colonization [23]. In our study, HT treatment induced differential expression of 3449 genes and significantly altered the metabolite profile of HS2-S-26. Although the direct addition of HT to the medium warrants caution regarding potential carryover of plant-derived compounds, thorough washing of mycelia and the absence of key metabolites (including dauricine) in HT-only controls (Wu et al., unpublished data) [29] support a fungal origin for the observed changes. These findings establish integrated omics as a powerful strategy for deciphering the regulatory networks governing endophyte–host metabolic interactions.

HT elicitor can significantly promote the biosynthesis of secondary metabolites such as alkaloids, terpenoids, phenolic acids, and flavonoids, among which alkaloids (25.64%) and terpenoids (28.21%) are the two most abundant categories. The ten upregulated alkaloids identified belong to six distinct structural classes, including plumerane, phenolamine, pyridine, and isoquinoline types. Among them, dauricine, a representative benzylisoquinoline alkaloid, possesses significant pharmacological activities [48], although additional studies will be necessary to confirm its complete biosynthetic origin in the fungus. Its biosynthesis initiates with the Pictet-Spengler condensation of dopamine and 4-hydroxyphenylacetaldehyde (4-HPAA) to form (S)-norcoclaurine, the universal precursor of all benzylisoquinoline alkaloids [56]. Consistent with this biosynthetic route, multiple genes (e.g., *AROF*, *TYR1*, *GOT2* and *ARO8*) involved in tyrosine biosynthesis were transcriptionally upregulated. The coordinated upregulation of the key shikimate pathway enzymes AROF (DAHP synthase) and ARO8 (aromatic aminotransferase) was associated with enhanced tyrosine metabolism, which may contribute to the precursor supply of dopamine for subsequent dauricine biosynthesis. All gene functional assignments are correlative and require functional validation to confirm causality. However, genes directly responsible for the downstream conversion of tyrosine-derived precursors to dauricine, including norcoclaurine synthase, O-methyltransferases, and cytochrome P450s implicated in oxidative coupling, were not significantly upregulated or could not be confidently annotated in our transcriptomic dataset [57]. This suggests that the biosynthetic pathway of dauricine in this fungus may involve distinct enzymes with low sequence similarity to known plant counterparts [58]. These basic findings are consistent with research showing that precursor supply modulates alkaloid accumulation [59,60].

Many studies have shown that terpenoids are biosynthesized via the MVA and methylerythritol phosphate (MEP) pathways [52]. In strain HS2-S-26, terpenoids appear to be primarily biosynthesized via the MVA pathway, as HT treatment upregulated key MVA genes (*MVAK2*, *GGPS1*, *FDFT1*) while downregulating ubiquinone branch genes *COQ2* and *WRBA* (NAD(P)H dehydrogenase) (Table S9). Additionally, the downregulation of *CYP505* in diterpene biosynthesis, coupled with the marked accumulation of isopimaric acid, suggests a possible association between reduced hydroxylation and product accumulation, a phenomenon also observed in the taxol biosynthetic pathway where decreased CYP450 activity has been associated with diterpene intermediate accumulation [61]. These observations suggest that HT may coordinate terpenoid biosynthesis through a combination of core pathway activation and branch pathway suppression, potentially redirecting metabolic flux toward terpenoid production. This regulatory pattern is consistent with previous reports [53], supporting the view that host-derived substances can promote secondary metabolite biosynthesis in endophytic fungi, possibly by activating precursor pathways or silent some gene clusters. These findings provide a scientific basis for elucidating the molecular mechanisms of host–endophyte interactions, and suggest a possible strategy for applying co-culture approaches to enhance the production of target secondary metabolites.

## 5. Conclusions

111 endophytic fungal strains were isolated from wild *H. serrata*, among which 51 strains exhibited AChE inhibition rates exceeding 50%, and 18 strains exceeded 90%. Strain HS2-S-26, with strong activity (98.37%), was further investigated. HT (*H. serrata* thallus) was used as a host-derived elicitor to modulate fungal metabolism. Dauricine, a pharmacologically active benzylisoquinoline alkaloid, was detected in HS2-S-26. Transcriptomic and metabolomic analyses revealed that HT upregulated key genes in terpenoid (MVA) and tyrosine biosynthesis, enhancing alkaloid (including dauricine) and terpenoid accumulation. However, the correlative nature of gene functional assignments should be acknowledged, and future studies should focus on genome sequencing and functional validation of candidate genes. These findings support host thallus as an effective elicitor and offer a multi-omic perspective on host–endophyte metabolic regulation.

## Figures and Tables

**Figure 1:**
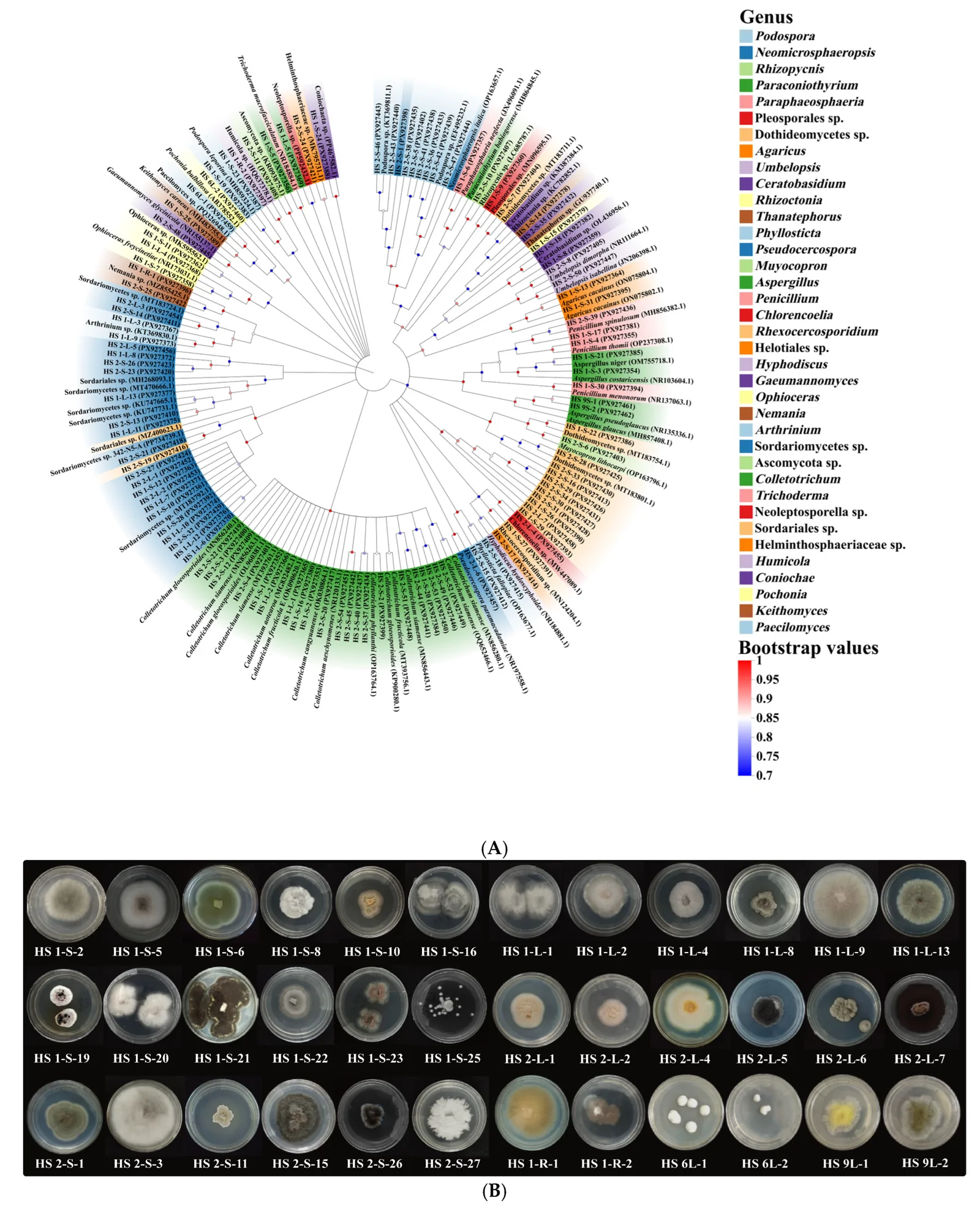
Endophytic fungi isolated from *H. serrata*. (**A**) Neighbor-Joining (NJ) Phylogenetic tree based on ITS sequences of endophytic fungi isolated from *H. serrata*. Species names or culture collection numbers are followed by GenBank accession numbers in parentheses. Bootstrap support values greater than 0.7 from 1000 replicates are visualized at the nodes using a gradient color scale. (**B**) Colony morphologies, growth characteristics on PDA plates.

**Figure 2:**
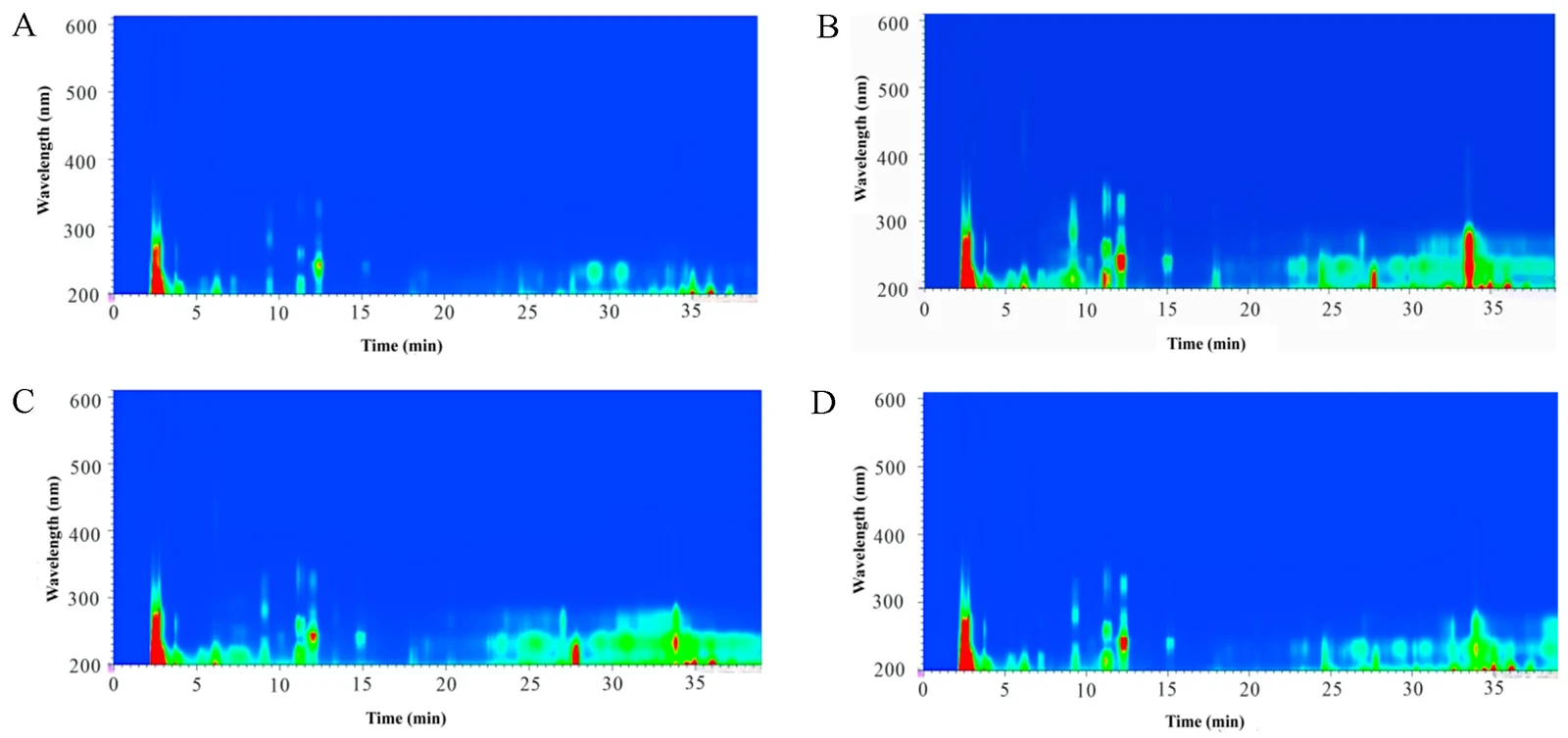
Multi-wavelength HPLC analysis of co-fermentation crude extracts. (**A**) Control group without HT elicitor addition. (**B**–**D**) Experimental groups supplemented with 1, 2, or 3 g/100 mL of HT elicitor. The color scale represents metabolite abundance, with red indicating high-abundance metabolites.

**Figure 3:**
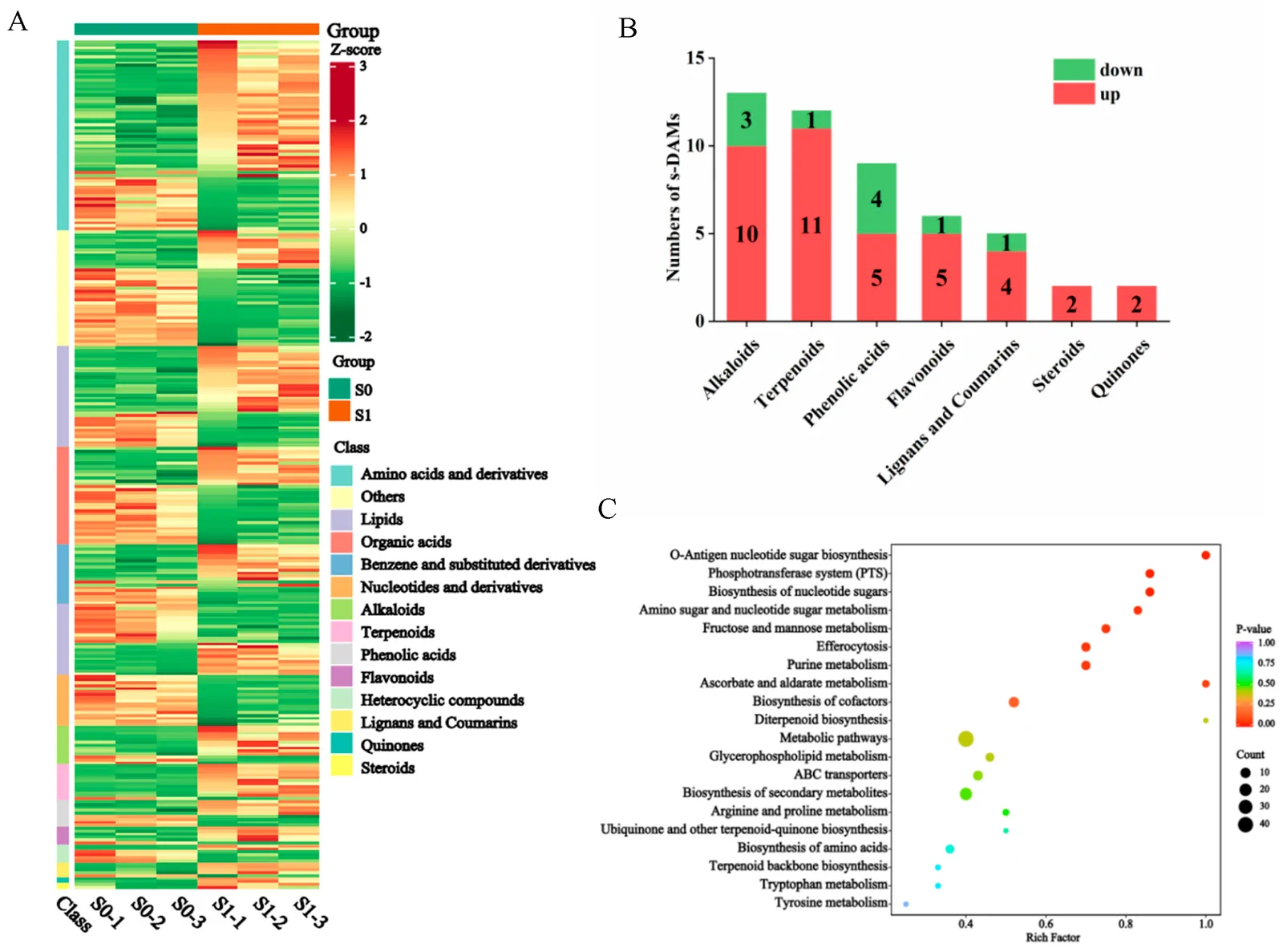
Analysis of differential metabolites. (**A**) Heat map of all DAMs between the S0 and S1 groups. (**B**) Quantitative profile of differential secondary metabolites. (**C**) KEGG pathway enrichment bubble diagram of total DAMs. The ordinate and abscissa represent the KEGG pathway and the enrichment factor. The bubble color intensity indicates the statistical significance, and a larger rich factor denotes stronger enrichment.

**Figure 4:**
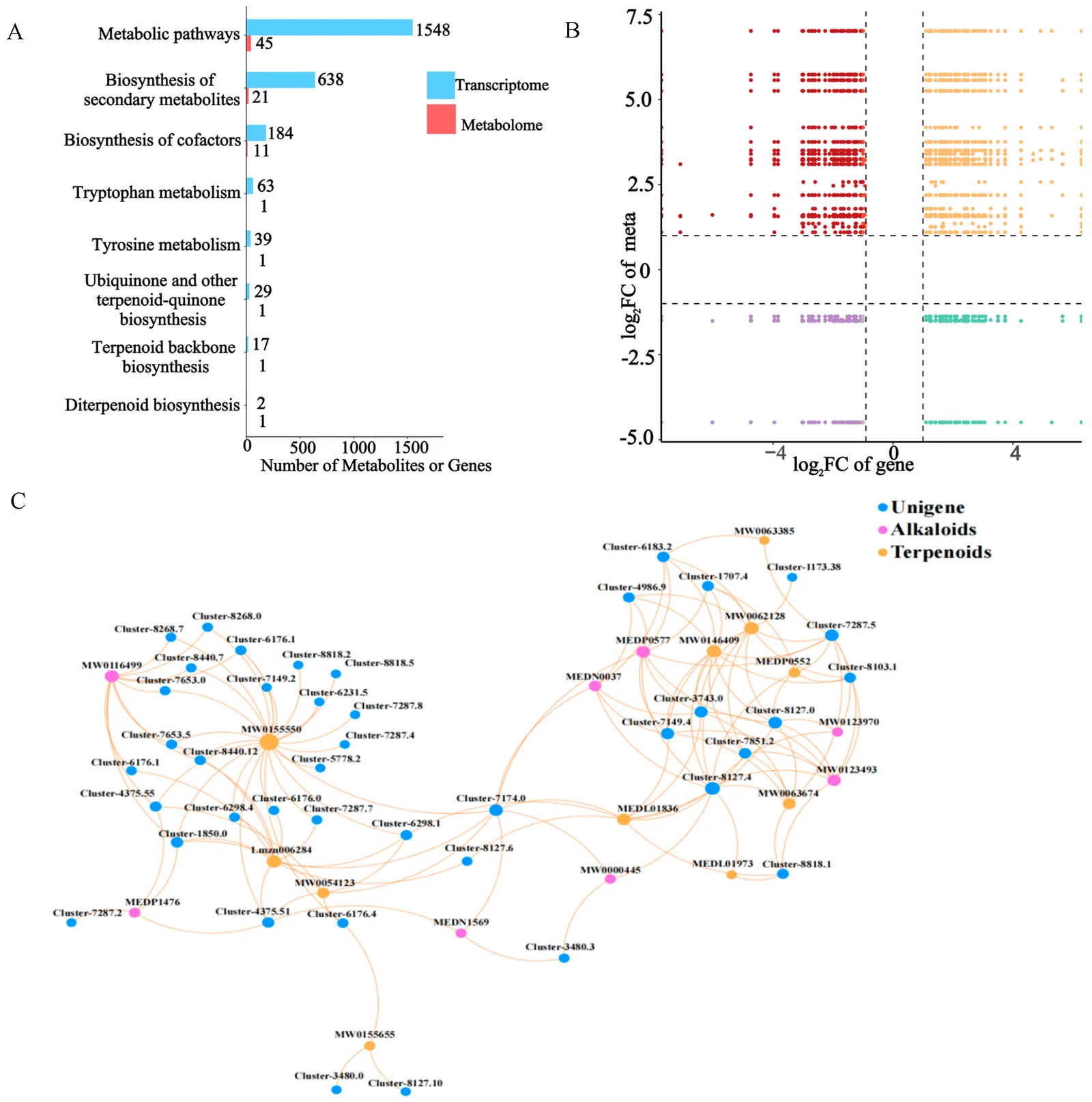
Correlation analysis between differentially accumulated secondary metabolites (s-DAMs) and DEGs. (**A**) KEGG enrichment analysis of DEGs (blue column) and s-DAMs (red column) enriched in the same pathway. (**B**) Nine-quadrant plot of Pearson’s correlation between transcriptomic and metabolomic datasets. Significant correlations were defined as |PCC| > 0.8 and *p* < 0.05, with dashed lines at ±1 log_2_FC on both axes. Orange (upper-right) and purple (lower-left) points represent features consistently up- or downregulated across datasets, whereas red (upper-left) and green (lower-right) points indicate opposite regulatory trends. (**C**) Correlation network analysis of terpenoid and alkaloid biosynthesis (PCC > 0.8, *p* < 0.01). The three colored circles represent unigenes, alkaloids, and terpenoids, respectively. Larger circles indicate a greater number of associations.

**Figure 5:**
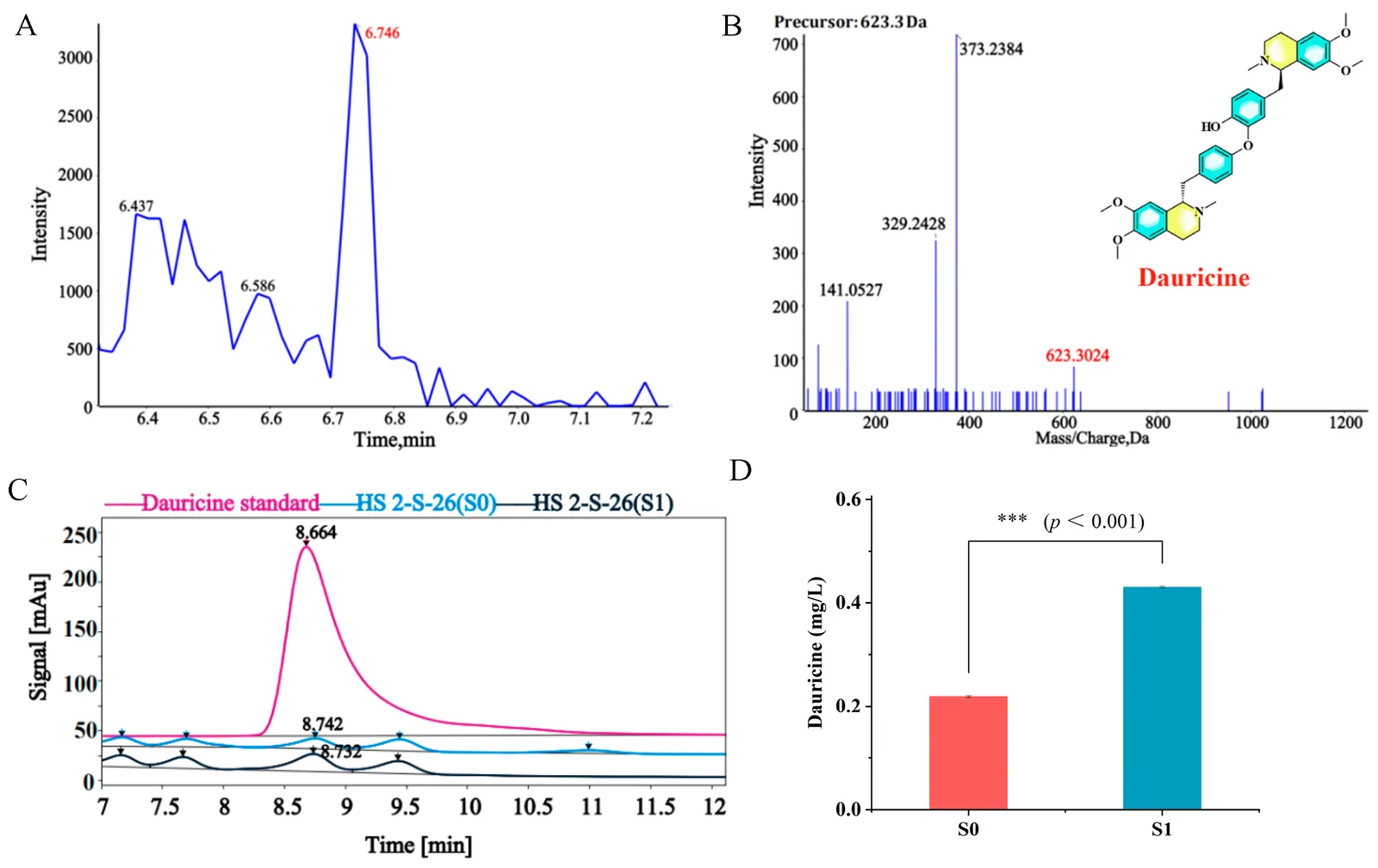
Detection of dauricine in HS2-S-26 by UPLC-MS/MS and HPLC. (**A**,**B**) UPLC-MS/MS analysis of dauricine ([M-H]^−^ = *m*/*z* 623.3024) produced by the strain. (**C**) HPLC analysis of the strain (S0, S1) was performed at 284 nm, with dauricine standard as control. (**D**) Dauricine content in S0 and S1 groups. Values are presented as the mean ± standard deviation (SD) of three replicates. The “***” indicates a statistically significant difference in content between the two groups (*p* < 0.001).

**Figure 6:**
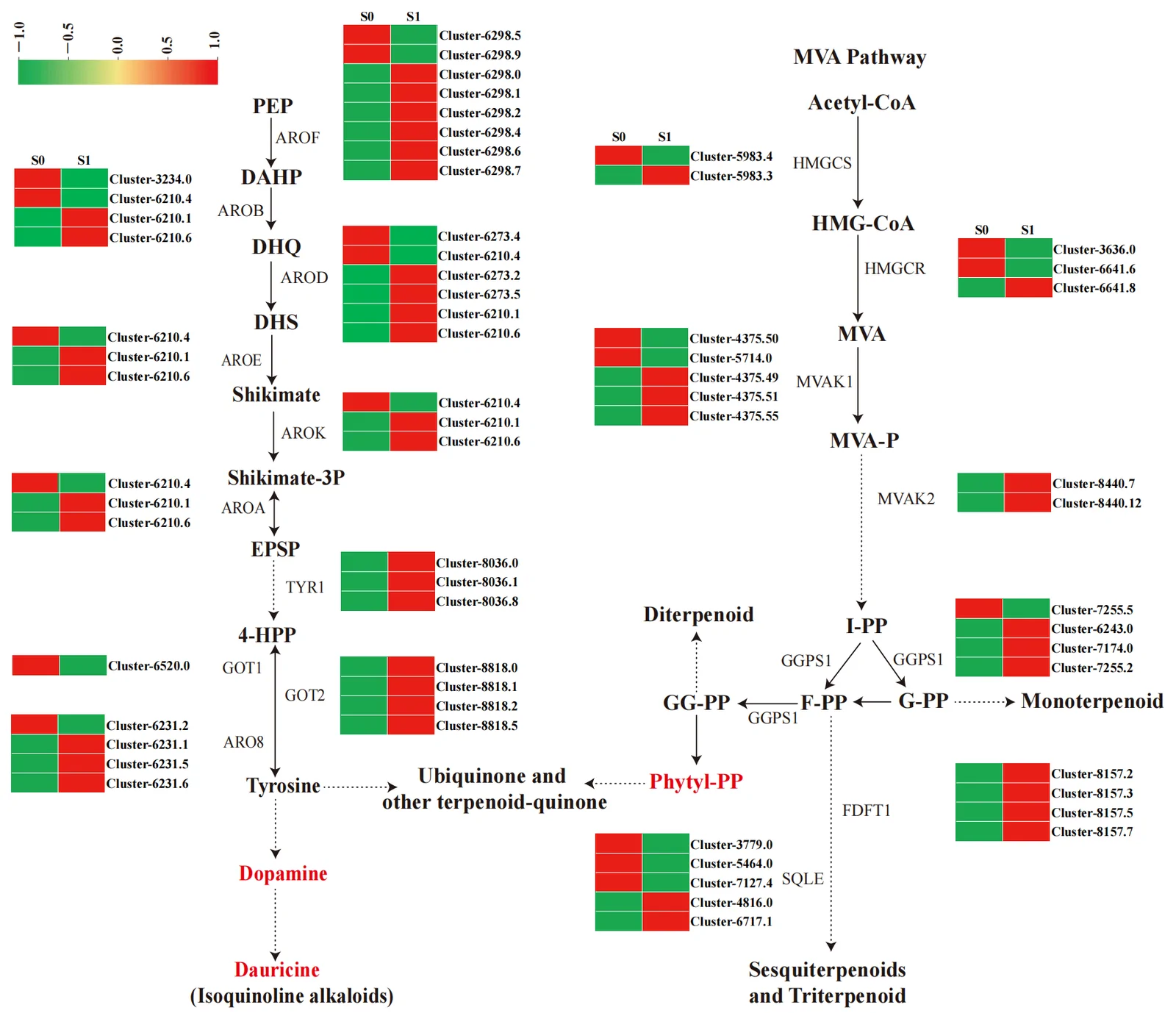
Pathways and genes involved in dauricine and terpenoid biosynthesis in HS2-S-26. Expression levels of enzyme genes are depicted as heatmaps in the colored cells positioned on the left or right side; green shows downregulation, and red shows upregulation. Metabolites highlighted in red are those that have been detected and show significant upregulation in S1. Solid arrows indicate that the reaction leads directly to the indicated compound, whereas dashed arrows indicate that the reaction involves intermediate steps that are not shown (unlabeled) in the figure.

**Table 1:** qRT-PCR primer sequence.

Target Genes	Forward Primer Sequence (5′-3′)	Reverse Primer Sequence (5′-3′)
β-tubulin	CCGTGGTGCTCACTCTTTC	TCCTCAACCTCCTTCATCG
Cluster-3636.0	CCGCAGAACTTGTCGGTCAT	GGTCGTCGGTCGGGATTT
Cluster-3234.0	GGGATTACCAACGTCAAGCT	GACCACCACCAACAACCAG
Cluster-7174.0	TGACGGCCATGTCCAACAAC	TGTAGACGGCGAGCAGGGAG
Cluster-8818.1	CTGCTGAAGAAGAACCTGGAGA	TGGTGGCATACACGCTGTG
Cluster-8157.2	GCGAGCTTCAGCAACTTCG	CGCTCAGCGTGTTCGTGT
Cluster-4375.55	CAGTATGCCTCGACAACGAA	CTGGGACCCACTTGATCTTT
Cluster-4375.49	GAGTTGCCGGGATTGTTAC	CGATTGAGCCCAGTTGAG
Cluster-5983.3	TGGAGCCACGACGAAGAC	CGACATAAACGCCGACTG
Cluster-7707.1	CTCGATTACCCTGAACGTGTCG	CAAGAACCCGTCGATGATGC

**Table 2:** Alkaloids and terpenoids with significant differences in the S1 vs. S0 group.

Index	Compounds	Formula	Class	VIP	log_2_FC
MEDP0251	Trigonelline	C_7_H_7_NO_2_	Alkaloids	1.28	−1.48
MEDL01940	Feruloylputrescine	C_14_H_20_N_2_O_3_	Alkaloids	1.32	−4.49
MEDP1476	5-Methoxytryptamine	C_11_H_14_N_2_O	Alkaloids	1.13	1.26
MEDP0577	Carnitine isoC4:0	C_11_H_21_NO_4_	Alkaloids	1.35	1.56
MEDP1315	Quinoline	C_9_H_7_N	Alkaloids	1.31	−1.51
MEDN0037	Dopamine	C_8_H_11_NO_2_	Alkaloids	1.26	1.10
MW0000445	4,12-Dimethoxy-17-methyl-13- oxo-17-azoniatetracyclo [7.5.3.01,10.02,7]heptadeca- 2(7),3,5,11-tetraen-3-olate	C_19_H_23_NO_4_	Alkaloids	1.39	7.02
MW0123493	Dauricine	C_38_H_44_N_2_O_6_	Alkaloids	1.37	3.10
MW0149512	Geissospermine	C_40_H_48_N_4_O_3_	Alkaloids	1.39	5.57
MEDN1569	Piperlongumine	C_17_H_19_NO_5_	Alkaloids	1.01	2.47
MEDL01793	Jubanine A	C_40_H_49_N_5_O_6_	Alkaloids	1.37	5.25
MW0123970	Ethyl 3-isothiocyanato-1H- indole-2-carboxylate	C_12_H_10_N_2_O_2_S	Alkaloids	1.13	1.36
MW0116499	1-(cyclohexylamino)-3-(6-methy-1,2,3,4-tetrahydro-9H- 2-carbazol-9-yl)propan-2-ol	C_22_H_32_N_2_O	Alkaloids	1.21	1.79
MW0062128	Polyporusterone A	C_28_H_46_O_6_	Terpenoids	1.36	3.25
MEDP0552	Alantolactone	C_15_H_20_O_2_	Terpenoids	1.24	4.18
Lmzn006284	Corosolic acid	C_30_H_48_O_4_	Terpenoids	1.37	1.58
MW0054123	Isopimaric acid	C_20_H_30_O_2_	Terpenoids	1.27	1.61
MW0063674	Steviobioside	C_32_H_50_O_13_	Terpenoids	1.30	3.40
MW0063385	Rubusoside	C_32_H_50_O_13_	Terpenoids	1.28	3.21
MW0155655	Polyphyllin B	C_51_H_82_O_20_	Terpenoids	1.09	2.58
MEDL01836	Capsianoside IV	C_32_H_52_O_13_	Terpenoids	1.36	2.19
MEDL01973	Maslinic acid 3-O-b-D-glucoside	C_36_H_58_O_9_	Terpenoids	1.40	5.73
MEDL02585	Veranisatin B	C_16_H_20_O_9_	Terpenoids	1.22	−1.38
MW0146409	Atractyloside D	C_27_H_46_O_12_	Terpenoids	1.38	3.50
MW0155550	Phytyl diphosphate	C_20_H_42_O_7_P_2_	Terpenoids	1.40	3.75

## Data Availability

The ITS sequences of endophytic fungal strains isolated in this study were deposited in the GenBank database under accession numbers PX927352-PX927462. Raw transcriptomic sequencing data were publicly available in the Genome Sequence Archive (GSA: CRA046477) via the National Genomics Data Center (https://ngdc.cncb.ac.cn/gsa accessed on 15 July 2026)). Raw metabolomic data were submitted to the OMIX database at NGDC with accession OMIX018657.

## Supplementary Materials

The following supporting information can be downloaded at: https://www.mdpi.com/article/10.3390/jof12100719/s1, Figure S1: Maximum likelihood (ML) phylogenetic tree based on *rpb2* gene sequences. Figure S2: Microscopic morphological features of representative endophytic fungal isolates from *Huperzia serrata*. Figure S3: HPLC detection of HupA in the fermentation products of strain HS2-S-26 with reference to the HupA standard. Figure S4: Principal component analysis of metabolomic data. Figure S5: OPLS-DA permutation test (200 permutations) for S1 vs. S0. Figure S6: Sequence length distribution statistics of the transcriptome assembly. Figure S7: Annotation statistics of transcriptomic unigenes in public databases. Figure S8: Venn diagram of KEGG pathway co-enrichment of DEGs and DAMs in integrated transcriptomic and metabolomic analysis. Figure S9: qRT-PCR validation of nine selected differentially expressed genes. Table S1: Identification results of endophytic fungi from *Huperzia serrata* (*H. serrata*). Table S2: Reference strains and GenBank accession numbers used for *rpb2* phylogenetic analysis. Table S3: AChE inhibition ratio of crude extracts of endophytic fungi from *H. serrata*. Table S4: A list of S1 vs. S0 DAMs. Table S5: Summary of RNA-seq in S1 and S0 groups. Table S6: The transcriptome assembled a total of 19,329 unigenes. Table S7: DEGs KEGG enrichment analysis. Table S8: KEGG enrichment analysis of DEGs and DAMs. Table S9: FPKM values of key KEGG pathways (ko00350, ko00380, ko00130, ko00900, ko00904, and ko00400). Table S10: Pearson correlation analysis between DAMs and DEGs involved in the alkaloid and terpenoid biosynthetic pathways (PCC > 0.8, *p* < 0.01). Table S11: Unigenes involved in the alkaloid and terpenoid biosynthetic pathways (PCC > 0.8, *p* < 0.01).

## Abbreviations

The following abbreviations are used in this manuscript:

4CL	4-coumarate-CoA ligase
4-HPP	4-Hydroxy-phenylpyruvate
ALDH	Aldehyde dehydrogenase (NAD^+^)
ALDH8A1	Aminomuconate-semialdehyde dehydrogenase
ARO8	Aromatic amino acid aminotransferase I/2-aminoadipate transaminase
AROA	3-phosphoshikimate 1-carboxyvinyltransferase
AROB	3-dehydroquinate synthase
AROD	3-dehydroquinate dehydratase I
AROE	Shikimate dehydrogenase
AROF	3-deoxy-7-phosphoheptulonate synthase
AROK	Shikimate kinase
COQ2	4-hydroxybenzoate polyprenyltransferase
DAHP	7P-2-Dehydro-3-deoxy-D-arabino-heptonate
DHQ	3-Dehydroquinate
DHS	3-Dehydroshikimate
EPSP	5-O-(1-carboxyvinyl)-3-phosphoshikimate
FAHD1	Acylpyruvate hydrolase
FDFT1	Farnesyl-diphosphate farnesyltransferase
F-PP	Farnesyl-diphosphate
GG-PP	Geranyl geranyl diphosphate
GGPS1	Geranylgeranyl diphosphate synthase, type III
GOT1	Aspartate aminotransferase, cytoplasmic
GOT2	Aspartate aminotransferase, mitochondrial
G-PP	Geranyl diphosphate
HGD	Homogentisate 1,2-dioxygenase
HMGCR	3-hydroxy-3-methy-lglutary-CoA reductase
HMGCS	3-hydroxy-3-methylglutaryl-CoA synthase
INDO	Indoleamine 2,3-dioxygenase
I-PP	Isopentenyl diphosphate
MVA	Mevalonate
MVAK1	Mevalonate kinase
MVAK2	Phosphomevalonate kinase
PEP	Phosphoenolpyruvate
SQLE	Squalene monooxygenase
TRP	Tryptophan synthase
TYR1	Prephenate dehydrogenase (NADP^+^)
WRBA	NAD(P)H dehydrogenase
